# Costs of molecular adaptation to the chemical exposome: a focus on xenobiotic metabolism pathways

**DOI:** 10.1098/rstb.2022.0510

**Published:** 2024-03-25

**Authors:** Céline Tomkiewicz, Xavier Coumoul, Pierre Nioche, Robert Barouki, Etienne B. Blanc

**Affiliations:** ^1^ Université Paris Cité, Inserm unit UMRS 1124, 75006 Paris, France; ^2^ Hôpital Necker Enfants malades, AP-HP, 75006 Paris, France

**Keywords:** xenobiotic metabolism, aryl hydrocarbon receptor, microbiome, toxicology, epigenetics

## Abstract

Organisms adapt to their environment through different pathways. In vertebrates, xenobiotics are detected, metabolized and eliminated through the inducible xenobiotic-metabolizing pathways (XMP) which can also generate reactive toxic intermediates. In this review, we will discuss the impacts of the chemical exposome complexity on the balance between detoxication and side effects. There is a large discrepancy between the limited number of proteins involved in these pathways (few dozens) and the diversity and complexity of the chemical exposome (tens of thousands of chemicals). Several XMP proteins have a low specificity which allows them to bind and/or metabolize a large number of chemicals. This leads to undesired consequences, such as cross-inhibition, inefficient metabolism, release of toxic intermediates, etc. Furthermore, several XMP proteins have endogenous functions that may be disrupted upon exposure to exogenous chemicals. The gut microbiome produces a very large number of metabolites that enter the body and are part of the chemical exposome. It can metabolize xenobiotics and either eliminate them or lead to toxic derivatives. The complex interactions between chemicals of different origins will be illustrated by the diverse roles of the aryl hydrocarbon receptor which binds and transduces the signals of a large number of xenobiotics, microbiome metabolites, dietary chemicals and endogenous compounds.

This article is part of the theme issue ‘Endocrine responses to environmental variation: conceptual approaches and recent developments’.

## 1. The exposome and the eco-exposome

In the last few decades, much work has been done on the characterization of the genomes of different species (animals, plants, fungi, etc.) and on its biological and health implications. Less knowledge was acquired on environmental exposures and their biological impacts. This led Chris Wild in 2005 to put forward the concept of the exposome which was defined as the life-course environmental exposures (including lifestyle factors), from the prenatal period onwards [1]. With this definition, the exposome appears as the complement of the genome, and their interaction accounts for physiological and pathological states [2]. The exposome definition by Wild includes two important dimensions: (i) the exposome covers a variety of exposures including chemical, physical, biological, psychological, social and behavioural exposures; (ii) the exposome spans the life-course, including critical periods of vulnerability such as the prenatal period, newborns, puberty, the elderly. Additional contributions to the exposome concept aimed at the operationalization of the concept. Several authors highlighted the chemical component of the exposome, including Rappaport and Smith who focused on the thorough analytical characterization of chemicals in body fluids [3]. High-resolution mass spectrometry (HRMS) has been instrumental in characterizing the complexity of the exposure profile as well as the endogenous metabolome, which at least partially reflects the complexity of biological responses to environmental exposures [4,5]. Using these technologies, it is possible to study simultaneously thousands of compounds. Recently, this large-scale characterization, also known as exposomics, has been further integrated with the other omics (e.g. metabolomics, transcriptomics) leading to the concept of functional exposomics which is defined as the biological translation of the exposome, much as functional genomics refer to the functional expression of the genome [6,7]; this biological translation refers for example to the mechanisms of toxicity triggered by multiple exposures (e.g. psychosocial stress and chemical stress). While much of the interest in the exposome concept came from scientists involved in human health, a more global perspective was provided with the eco-exposome defined as the interactions and bidirectional influences between humans and ecosystems and giving more focus on the impact of environmental stressors on living organisms in ecosystems [8]. The eco-exposome provides additional opportunities for interactions between the ecotoxicological and human health communities and in many cases the questions and the approaches are similar.

Much of the effort in the exposome field has been devoted to its characterization and to the development of methodologies to measure the exposome and its complexity. The chemical component of the exposome has been particularly studied since it is now possible to analyze biological samples (blood, urine, hair, etc.) and gather data on thousands of chemicals and their degradation products using non-targeted screening methodologies. New questions can now be addressed in this field, for example understanding the impacts of complex chemicals mixtures on human health [9]. Indeed, the impact of mixtures has been highlighted early on in the pharmacological field referring to potential interactions between several drugs; regarding pollutants, although the mechanisms of biological adaptation to exposure to single chemicals have been studied for a long time [10], the challenge that the complexity of the exposome represents to these adaptive mechanisms remains poorly understood. This is mainly due to the huge diversity of chemicals we are exposed to; indeed, the chemical exposome does not only cover traditional xenobiotics such as contaminants, pollutants and drugs but it also includes dietary components and, importantly, microbial metabolites [4]. The aim of this review is to analyze these challenges and to identify the costs of such adaptive mechanisms when thousands of chemicals are considered instead of chemicals taken individually. It will be focused primarily on molecular adaptation mechanisms related to the xenobiotic metabolism pathways (XMP).

## 2. The biological adaptation to exogenous chemicals

**Figure 1.**
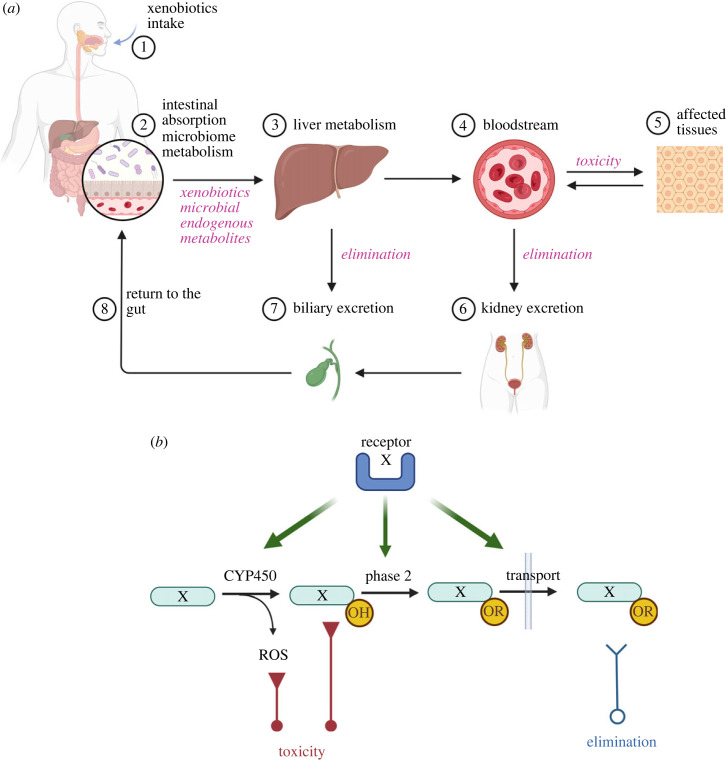
Fate of xenobiotics in mammalian organisms. (*a*) Following ingestion, xenobiotics can interact with the microbiome and can change its composition and/or be metabolized. Xenobiotics, their metabolites, as well as microbiome metabolites are absorbed and undergo further metabolism in the liver and possibly in other organs. Following this step, they can be eliminated in urine or, for some chemicals, in bile. Part of the latter chemicals are reabsorbed by the intestine. Before their elimination, some xenobiotics or their metabolites or microbial metabolites can elicit toxicity in different organs and tissues. (*b*) The xenobiotic-metabolizing pathways consist of different phases. Phase 1 is mostly composed of cytochromes P450 (CYP450) and consists of adding a reactive oxygen to mostly hydrophobic chemicals. Phase 2 consists of a variety of enzymatic reactions and leads to either detoxification or addition of a highly hydrophilic group (sugar, amino-acid, etc.), thus increasing water solubility of the xenobiotic metabolite. The latter is transported across the plasma membrane (phase 3), circulates in blood and is excreted in the kidney. An alternative excretion is through the bile. Importantly, some intermediates following CYP450 catalysis are extremely reactive with DNA and proteins and can cause toxicity. Furthermore, CYP450 activity leads to the release of reactive oxygen species that can also be toxic. Thus the detoxification pathway can also lead to some degree of toxicity, in particular during chronic exposure. X, xenobiotic.

Cells and organisms have developed defence systems that allow them to either detoxify or eliminate exogenous chemicals called xenobiotics. These defence systems are critical for cell (or population) survival and are found in all phyla, albeit with a large heterogeneity [11,12]. While long-term adaptation to external stressors relies on genetic or epigenetic mechanisms that can have multi-generational and evolutionary effects, we will focus here on short-term adaptation which relies on inducible systems that tend to limit the intensity or the effects of these stressors. In mammals, xenobiotics or their metabolites are excreted by the kidney or through the bile; they can be metabolized primarily in the liver as well as in other organs and for some of them by the gut microbiota (figure 1*a*). The major pathways involved in the short-term response to chemical stress is the inducible elimination and detoxication of xenobiotics which is carried out by xenobiotics metabolizing enzymes (XMEs) and transporters. XMEs contribute to the detoxification of xenobiotics and/or to the increase in their hydrophilicity ultimately leading to their excretion out of the organism (figure 1*b*). Indeed, many xenobiotics are hydrophobic compounds and would remain stored in fatty tissues without the activity of XMEs and transporters [13,14].

Interestingly, most of these defence systems are inducible and therefore adaptive, i.e. their activity is regulated according to a defined chemical environment [11]. Thus, in addition to the metabolic enzymes and transporters, the cellular response to xenobiotics includes xenobiotics receptors which are sensors for these chemicals that trigger the biological defence responses, i.e. the activity of XMEs and transporters [15]. While xenobiotics can bind to a variety of cellular receptors including hormone receptors, only a few of these receptors are involved in the XMPs because they are able to induce the metabolic pathways leading to xenobiotic elimination. These receptors are the aryl hyrocarbon receptor (AhR), pregnane X receptor (PXR), constitutive androstane receptor (CAR) and peroxisome proliferator-activated receptor *α* (PPAR*α*) [16,17]. Regulation of XMEs is a relatively short-term molecular response to the exposure to chemicals; it is possible that longer term adaptation mechanisms involving epigenetic regulation also contribute [18], but these have been less studied until now and they will be discussed below in a dedicated chapter.

To illustrate the metabolism of xenobiotics in mammals, we will discuss here the AhR pathway [15,19]. A large number of polyaromatic hydrocarbons (PAH) or polyhalogenated aromatic hydrocarbons bind the AhR and induce specific XMEs and transporters such as the phase 1 enzymes cytochromes P450 1A and 1B (which usually add an oxygen to hydrophobic molecules allowing further detoxifying activities), several phase 2 enzymes such as NAD(P)H quinone dehydrogenase 1, uridine-diphospho-glucuronosyltransferases and glutathione-S-transferases (tending to detoxify or to increase hydrophilicity by adding a highly hydrophilic chemical group) as well as membrane transporters. The function of this coordinated XMP (from the sensor to the effectors) is to increase the hydrophilicity of hydrophobic chemicals thereby considerably increasing their elimination rate and to detoxify certain xenobiotic metabolites (figure 1*b*) [14]. Xenobiotic metabolism is therefore an adaptive stress pathway that is triggered upon exposure to those chemicals. Importantly, these PAHs are not only related to human activities but they can also be generated from forest fires, volcanic activities and other natural or human activity involving combustion. Thus, these organisms use pathways that metabolize xenobiotics of natural origins to detoxify man-made substances which have increased considerably since the industrial revolution.

This elegant process is far from being perfect and surprisingly, the XMPs have also been shown to ‘bioactivate’ certain xenobiotics, notably procarcinogens, and to increase their toxicity [20].

These paradoxical effects stem from the biochemical reactions needed to eliminate xenobiotics; indeed, in order to add a hydrophilic group to hydrophobic xenobiotics such as the PAHs present in polluted air and in tobacco smoke, it is first required to add a reactive oxygen (phase 1 metabolism) leading to a first class of intermediate (e.g. epoxides or hydroxyls). In the case of an added epoxide, it is rapidly transformed to hydroxyl derivatives that can support the addition of other hydrophilic groups (phase 2 metabolism) [21]. However, these epoxide intermediates are extremely reactive and thus can lead to a variety of toxic effects such as DNA adducts (xenobiotic metabolites covalently bound to DNA), which may ultimately lead to mutations [22]. Furthermore, the activity of cytochrome P450 is known to release reactive oxygen species which may also lead to toxicity. As will be discussed later, a good balance between the different enzymes of these pathways can limit the risk of intermediate accumulation [14]. However, there are gene variants of these enzymes that may result in higher concentrations of these intermediate and deleterious effects. The production of these reactive intermediates as part of the XMPs illustrates the costs of molecular adaptation to the exposure to xenobiotic chemicals, since repeated activation of this pathway can stochastically lead to an increased risk of hazards [10]. This has been well characterized for some chemicals such as PAHs as well as other classes of chemicals. However, since the chemical exposome consists of thousands of chemicals to which we are exposed either simultaneously or successively, the implication for adaptive pathways and for the costs of such adaptative mechanisms are less understood.

## 3. The chemical exposome response pathways

A major input of the exposome approach is to consider exposures in a holistic way and in particular, concerning the chemical exposome, to take into consideration that organisms are exposed to a very large number of chemicals and not to a single one. The biological response associated with the chemical exposome takes into account the complexity and high dimensionality of the exposures. There are several biological systems that respond to highly complex signals consisting in very high numbers or even an infinity of entities and these include the xenobiotics response system, the olfactory system and, concerning large molecules, the immune system. Interestingly, these biological systems sensing high numbers of entities are in fact quite different. We will focus on the xenobiotic detection and response systems, the other two systems being discussed in box 1.

Box 1. **Olfactory and immune systems**

Odorants should actually be considered as part of the chemical exposome. The olfactory system is another biological system which is aimed at detecting environmental signals and in that case, there is a repertoire of hundreds of constitutive olfactory receptors and hundreds of corresponding genes. The combination of activation of several of these receptors provides an encoding mechanism for the detection of a very large number of odors [23].

Interestingly, the immune system, which is another biological system associated with complex signals, is quite different. Indeed, a very large repertoire of antibodies that display high specificity to non-self proteins can be produced through genetic rearrangements. In that sense, the ‘adaptive’ nature of the immune response is both quantitative (increase in the amount of specific antibodies) but also qualitative (specific increase in only few antibodies out of thousands of possibilities). This is quite different from the xenobiotics metabolizing and elimination system (limited number of proteins with poor specificity). Note, however, that the innate immune reaction is also limited to certain danger signals and displays little specificity [24].

The set of proteins involved in XMPs are meant to handle, in theory, an infinite number of possible xenobiotics (although in practice only those chemicals that may display a risk of toxicity are most relevant). With these numbers and taking into consideration the number of genes in humans (approx. 21000) and animals, it is impossible to have constitutive proteins of these pathways dedicated to a single or a small number of chemicals. Thus, several of the proteins involved in this system bind or metabolize a very large number of chemicals [25]. For example, in humans, there are around 20 xenobiotics-metabolizing CYPs and only a few bona fide xenobiotics receptors. Although the number of chemicals that are actual substrates of xenobiotics-metabolizing CYPs is extremely variable [26], some of them detect and respond to hundreds or thousands of chemicals, a property sometimes defined as promiscuity [27].

To address the possible costs of molecular adaptation to the xenobiotic component of the chemical exposome, the challenge is to understand how so few proteins can detect and respond to such a large number of substances. This is in fact related to the poor specificity displayed by these proteins. For example, the AhR can bind hundreds, maybe thousands of chemicals, likely due to the intrinsic properties of its ligand binding site [28,29]. The xenobiotic receptor PXR is also highly promiscuous [30,31]. Similarly, several CYPs also metabolize a fairly large number of chemicals (CYP1A1, CYP3A4) while others have a more restricted set of substrates [13]. The molecular basis for this poor specificity is likely to be structural in that many of these proteins have been shown to have large flexible ligand-binding pockets and to undergo ligand-induced conformational changes [32,33]. Thus, while in adaptive immunity, genetic recombination provides biological diversity, in the xenobiotic response system, protein structural flexibility may account for the adaptive capacity.

Xenobiotics are not the sole constituents of the internal component of the exposome. The latter is much wider and comprises chemicals from the microbiome and from diet. Some of these chemicals as well as some endogenous metabolites may be structurally similar to xenobiotics and may also be recognized by proteins of the xenobiotics response, in particular because of the poor specificity of the xenobiotics receptors and metabolizing enzymes. This makes the adaptation to the chemical exposome even more complex, with chemicals competing for the same binding sites, leading to several types of interactions (synergy, additivity, antagonism) that may trigger undesired toxic effects [28].

## 4. The implications of the poor specificity of the chemical exposome response system

There are several implications for the poor selectivity of the chemical exposome adaptive mechanisms that we will discuss below.

— **Inconsistent activities of the different components of the biological pathway**. This is best illustrated by the case of persistent organic pollutants (POPs) which are detected by the xenobiotic receptors but are not subsequently transformed by the XMEs leading to their bioaccumulation in fatty tissues. For example, the half-life of the Seveso dioxin (also named 2,3,7,8-tetrachlorodibenzo-p-dioxine or TCDD) in the human body exceeds 7 years. TCDD binds and activates the AhR but is not metabolized by the induced enzymes (CYP1 family). Indeed, biological systems are usually not capable of breaking halogen–carbon bonds. Thus, the pathway is activated but it is inefficient for the elimination of such a xenobiotic [10]. It can then metabolize other molecules at a higher rate and lead to oxidative stress (see below). This is the case of several halogenated compounds that tend to be persistent in humans for this reason, including dioxins, furans, polychlorinated biphenyls (PCBs), polybrominated flame retardants and per- and polyfluoroalkyl substances (PFAS), many of which are covered by the Stockholm convention [34].
— **Increased risk of uncoupled substrates**. The poor selectivity of the cytochromes P450 increases the risk of oxidative stress. Indeed, both xenobiotics and O_2_ are substrates of CYPs and, for the enzymatic reaction to take place, O_2_ is activated into a free radical that reacts with the xenobiotic. In some cases, the latter is not well positioned to be oxidized and both unmodified substrate and free reactive oxygen species are released in the medium [35]. Such xenobiotic substrates are called uncoupled substrates. The existence of very large numbers of xenobiotic substrates for CYPs increases the risk of inadequate positioning in the active site, therefore of uncoupling and of increased release of reactive oxygen species. This may result in toxic outcomes.
— **Inhibitory effects**. While TCDD is not readily metabolized by cytochromes P450, it can yet bind to some of them with high affinity (CYP1A2), thus leading to competition with other putative substrates [36,37]. This is particularly relevant since organisms are always exposed to complex mixtures which *per se* can lead to competition, but this is worsened by the pure inhibitory activities of certain substances such as dioxin.
— **Unintentional metabolism of endogenous substances**. The poor specificity of some components of the metabolism of xenobiotics pathway is such that endogenous compounds with structural similarity can also bind to these enzymes and be metabolized. This may be the case for estradiol which is metabolized by CYP1B1 into genotoxic catechol compounds, also leading to decreased hormone levels [38]. Thus, intrinsic properties of the xenobiotic interacting proteins, i.e. their promiscuity and poor selectivity which are important for their functions, are also responsible for toxic side effects that may lead to long-term toxicity.

## 5. The multiple physiological functions of the exposome response system components: the aryl hydrocarbon receptor as a model system

During the last few years, xenobiotics receptors and enzymes have been shown to be able to respectively bind and metabolize dietary, microbial and endogenous substances in addition to xenobiotics. It was initially thought that this was only due to the lack of specificity and consequently to errors made by the biological system (see above). However, in addition to the latter mechanism, it was found that several proteins of the xenobiotic response pathway may have multiple functions, including endogenous functions. We will illustrate this with the case of the AhR.

The most striking evidence that the AhR has other functions than sensing xenobiotics came from knock-out (KO) studies. AhR-/- mice display a number of defects in the vascular, liver, immune, reproductive and ocular systems in the absence of any xenobiotic, indicating that AhR has other endogenous functions [39,40]. In parallel, a significant amount of work has been devoted to the characterization of the AhR proteins in different species (sequence homology compared to humans or rodents, expression or not in various tissues, biological functions). Indeed, the AhR is an ancient protein that appeared in evolution with the eumetazoans (600 Ma) [41] but its function in vertebrates and invertebrates is different. As mentioned earlier, in vertebrates, the AhR was initially thought to be primarily involved in binding xenobiotics and triggering their elimination. Conversely, in invertebrates, the protein appears to be primarily involved in development and neuronal differentiation [15,42]; indeed, in *Drosophila*, the AhR is involved in development and organogenesis while in *Caenorhabditis* *elegans*, it regulates the differentiation of specific neurons. While it was initially thought that it was not activated by chemicals in the latter species, it was then discovered that it was activated or inhibited by a few chemicals (some hydrocarbons) but certainly not the same variety of substances as those of mammalian AhR [43]. This indicated that in some species, the primary functions of the AhR were related to development and to the functions of the nervous system, raising the possibility that some of these endogenous functions may have been conserved in vertebrate AhR, as was actually shown following the characterization of AhR KO mice. In addition, the AhR has been shown to be involved in myelin formation in both mice and humans [44]. An interesting case is that of fish, in particular zebrafish. The zebrafish actually have three versions of the AHR (called 1a, 1b and 2) due to gene duplication that occurred during evolution [41]. Each receptor has its own characteristics: AhR2 and AhR1a are expressed in a wider range of tissues than AhR1b; AhR2 is thought to have developmental or physiological functions that AhR1s do not have. Each receptor binds a variety of ligands (but different panels of chemicals, for example with TCDD binding only AhR1b and AhR2) [45]. Thus, over the course of evolution, the structure, the function and in some cases the number of the AhR genes has changed possibly to adapt to environmental changes and organism complexity.

**Figure 2.**
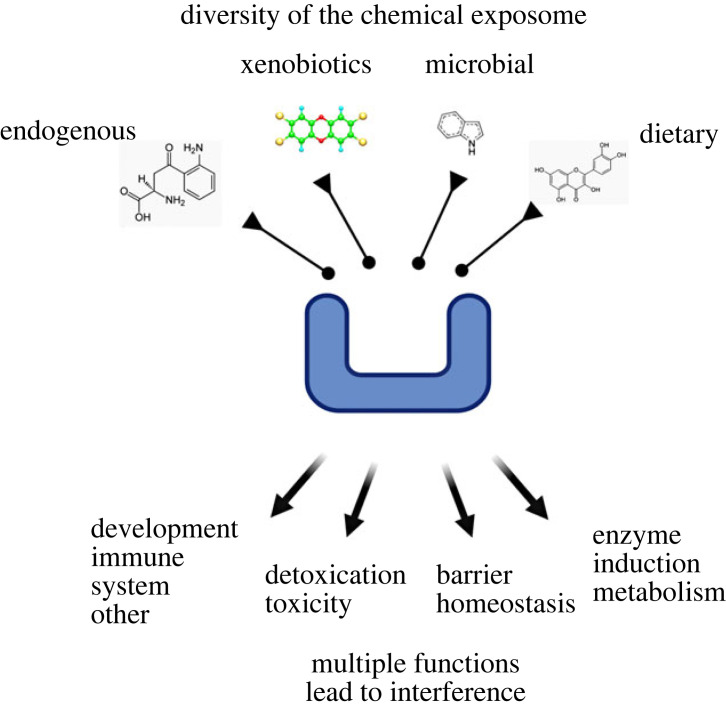
Aryl hydrocarbon receptor (AhR) ligands and functions. It is now well established that the AhR has a variety of ligands such as xenobiotics, dietary compounds, microbiome metabolites and endogenous substances. It consequently has a number of different functions in detoxification, the immune system and barrier organ homeostasis, as well as in the nervous system and in development. While this ‘one protein-many functions' condition may be related to evolution and possibly to ‘biological sobriety’, it also may lead to interference and to toxic consequences. This illustrates one aspect of the costs of adaptation to a highly complex chemical exposome.

Furthermore, other studies indicated that many microbial, dietary and endogenous substances were able to bind vertebrate AhRs, again suggesting a variety of functions (figure 2). It was also shown that depending on the type of chemicals, the biological effects of the AhR appear to be different, in particular the induced genes and the binding to DNA sequences [28]. Several laboratories have attempted to solve the structure of the AhR ligand binding domain using X-ray crystallography but failed, likely due to the high flexibility of the protein. Recently the structure of the AhR PAS-B domain in complex to HsP90 and XAP2 was obtained using cryo-electronic microscopy, paving the way for additional understanding of the mechanisms of ligand binding and activation [46]. *In silico* models and recent structural studies indicate that the invertebrates and vertebrates binding pockets are quite different, explaining also the different functions of the AhR in an evolutionary perspective [47].

A striking function of the AhR is its involvement in immune regulation in mammals and its presence in different immune cells [48]. Interestingly, the AhR is expressed in intestinal lymphocytes and appears to be involved both in the immune tolerance of certain bacteria of the microbiota and in the toxicity of other microbial species. Another function is related to the metabolism of tryptophan, since many metabolites of this amino acid bind the AhR and may have an effect on cell survival as well as other cellular functions [49].

Because of its multiple functions, it was recently suggested that the AhR represents an exposome receptor [28]. Importantly, other xenobiotic receptors also bind xenobiotic and endogenous metabolites. This is the case for the PXR which binds bile acids in addition to a variety of xenobiotics [50]. The consequences of a receptor having multiple functions are diverse. For example, if the AhR binds dioxin, it will not be able to bind microbial or endogenous substances and therefore, dioxin would not only trigger the xenobiotic response pathway, but also inhibit other pathways involving this receptor [51]. The inhibition of the physiological effects of the AhR is amplified by the induction of its repressor (AhRR) that will further down-regulate the activity of the receptor.

In conclusion the fact that some proteins of the chemical exposome response pathway display multiple functions may lead to competition with physiological processes and toxicity.

## 6. Long-term adaptation and epigenetics

**Figure 3.**
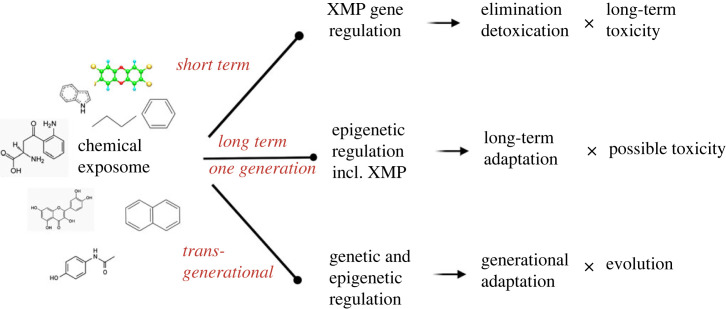
Adaptive mechanisms involved in the response to xenobiotics. Short term adaptation is primarily through the induction of XMPs which leads to detoxication and elimination, but which is also accompanied by toxicity that may be apparent in the long term. Long-term (one generation) adaptation may occur through epigenetic regulation, e.g. of XMP genes leading to detoxication and elimination. This my lead to toxic outcomes but the evidence is scarce at this stage. Transgenerational adaptation may also occur possibly through epigenetic mechanisms, but this has been less well studied at the moment.

In many species, long-term adaptation depends on genetic or epigenetic regulation and can drive evolution [52]. Epigenetic regulation leads to heritable changes in gene expression and is an important mechanism of long-term effects. In mammals, chemicals have been shown to elicit epigenetic effects in particular through the regulation of DNA methylation but also histone modifications and non-coding RNAs (figure 3) [53]. These changes in epigenetic marks have been found in a variety of tissues, including sperm, which may indicate possible transgenerational effects [54]. In humans, several epidemiological studies were able to correlate exposure to chemicals and changes in epigenetic markers, which may support current efforts towards precision prevention [55,56]. Most of the studies considered these epigenetic changes as possible markers of toxicity, in particular long-term toxicity [54]. Whether these changes could also be adaptive in mammals has been addressed in a few studies. In transgenerational studies, exposure to the vinclozoline fungicide of the F0 generation led to epigenetic changes that correlated with mate preference at the F3 generation. These data indicate that epigenetic changes may be associated with multigenerational adaptive behaviour [57].

Concerning XMPs, which are the focus of this review, it was shown that epigenetic mechanisms can influence the expression of XMP genes [58,59]. One of the best described cases is that of the long-term effect of the Constitutive Androstane Receptor (CAR) ligand TCPOBOP. This chemical is known to induce the Cyp2B10 and Cyp2C37 genes in mice through the activation of CAR. Exposure of mice in early life leads to a persistently induced expression of these two CAR target CYP genes with functional consequences [60]. This correlated with persistent modification of histones at the Cyp2B10 gene locus. These data indicate that epigenetic regulation of XMP genes is a mechanism for long-term adaptation to chemical exposure. Since the consequences of these regulations are increased expression of CYP genes, then it is likely that some of the side-effects of CYP upregulations described above will also be observed. At this stage, it seems important to further address these long-term intergenerational mechanisms to support these initial findings and to provide additional evidence for the adaptive roles of these epigenetic changes.

Studies on the epigenetic regulation of the AhR pathway have shown that the AhR gene as well as the AhR-target genes are regulated by epigenetic modifications, including DNA methylation, histone modification and miRNA [18]. These regulations were shown to be relevant for cell-type specific expression and in different disease states such as cancer [61,62]. Concerning the AhR target gene CYP1A1, it was shown that dioxin leads to a decrease in the methylation of its promoter [63]. This may suggest a sustained adaptive effect with possible adverse outcomes such as increased oxidative stress [64]. AhRR is another important AhR-target gene that is also regulated by epigenetic mechanisms [65]. CpG sites in the AhRR were shown to be differentially methylated in smoking individuals [66]. At this stage it is unclear whether these epigenetic modifications are related to the polyaromatic hydrocarbons present in tobacco smoke and whether this epigenetic regulation of AhRR is an adaptive response. However, hypomethylation of AhRR was associated with smoking-related morbidity [67].

Altogether, there are clear indications that the xenobiotic metabolic pathways are regulated by epigenetic mechanisms likely leading to long-term adaptive effects. The actual biological and/or toxic consequences of such regulations are still under investigation.

## 7. The multiple roles of the microbiota

There are several interactions between exogenous chemicals and the gut microbiome (GM) which are highly relevant for the composition of the chemical exposome and the body's response to such a challenge. Most of the studies in this field have focused on the health impacts of GM composition alterations following exposure to drugs or, to a lesser extent, to contaminants. An alternative approach to this problem would be to ask whether the GM plays a role in the response to the chemical exposome and what cost would this entail.

Microbiota metabolites are part of the so-called internal component of the chemical exposome [3]. By comparing conventional and germ-free mice, it was found that up to 10% of the blood metabolome originates or is influenced by the gut microbiome [68]. The actual origin of these metabolites is diverse; some originate purely from bacterial metabolites, others from bacterial metabolism of endogenous compounds (e.g. biliary acids or tryptophan metabolism), others from bacterial metabolism of dietary compounds (e.g. short chain fatty acids) or of xenobiotics including drugs and food contaminants. Altogether, organisms are exposed to a large variety of microbe-derived metabolites which thus influence health and disease. It is known that some microbial metabolites are beneficial to health [69], while others are toxic (e.g. trimethylamine N-oxide, TMAO produced by the liver from a bacterial metabolite, trimethylamine) [70]. There is also a very close interaction between the GM and the immune system.

The possible role of the GM in terms of adaptation to the chemical exposome raises a number of questions. The interactions between the GM and xenobiotics appears to be quite complex since these chemicals are metabolized by the GM, but they can also alter the GM composition with consequences on human health.

The GM contributes to the degradation of some dietary constituents such as fibres and this appears to be a contribution to digestion. Concerning xenobiotics, the role of the GM appears to be more complex. It is clearly involved in the degradation of xenobiotics (drugs, contaminants) and, considering the large diversity of enzymatic activities present in the GM, this activity could contribute to the elimination of xenobiotics. However, in some cases, microbial metabolism leads to degradation products that are more toxic than the parent compound. For example, melamine which was added to infant formula to artificially boost nitrogen content (a proxy for protein), is degraded by the GM into a highly toxic metabolite leading to severe human renal toxicity [71]. At this stage, it is possible to conclude that the GM contributes both to xenobiotic elimination as well as xenobiotic toxicity and, as such, it is part of the global system governing the response to the chemical exposome, in particular through the diversity of the enzymatic reactions that it harbours. However, it is unclear whether the global outcome of GM metabolic activities is protective or not. It is also unclear whether the GM can be considered as an adaptive component of the response to the chemical exposome and whether the induced changes in GM composition contribute to an improved response.

Xenobiotics, including drugs and contaminants, influence the GM composition and functions [72]. Antibiotics as well as other drugs and several contaminants are deleterious, but in other cases, the GM can contribute to the beneficial drug effects as in the case of metformin [73]. Yet, generally speaking, several xenobiotics alter GM composition which may influence an individual's health. In many cases, this can change the metabolites of microbial origin in human tissues, thus leading to additional changes in the internal component of the exposome.

The biology of the AhR has uncovered interesting links between the xenobiotics response system, the GM and the immune system. Indeed, in addition to its functions in sensing xenobiotics and triggering the adaptive response, the AhR has been shown to be expressed in the digestive tract lymph nodes and to have the capacity to sense microbial metabolites [48]. It thus plays a role in the immune tolerance of the bacteria present in the GM. Several microbiome-derived ligands of the AhR are implicated in such a function, including bacterial metabolites of tryptophane and bacterial virulence factors such as phenazine [74,75]. The function of the AhR in GM homeostasis has proven to be critical for the pathogenesis of diseases such as metabolic diseases and auto-immune diseases [76,77]. Clearly, the multiple roles of the AhR in xenobiotic sensing, in gut-microbiome homeostasis and in endogenous functions can lead to interferences between different functions and therefore to toxic outcomes.

## 8. Conclusion

In this article, we have discussed the properties of the XMPs and how they contribute both to the inducible elimination and detoxication of chemicals, but also to the long-term toxicity of some of these chemicals. The extreme diversity of the chemical exposome, coupled with the limited number of sensing and adaptive pathways, leads to a large number of interferences among xenobiotics but also between those chemicals and microbial or endogenous metabolites. As a consequence, these adaptive pathways are not only involved in the elimination and detoxification of exogenous compounds, but are also paradoxically involved in toxicity pathways. Obviously the structural or functional plasticity of these proteins seems sufficient to sense and trigger the elimination of a large number of chemicals but certainly not all of them. Furthermore, the additional functions of these receptors increase the risk of interferences with physiological processes leading to possible adverse outcomes. When specifically considering these XMPs, the capacity to adapt to the chemical exposome is accompanied by some toxic side effects, in particular when organisms are repeatedly exposed to high levels of chemicals.

While this review has focused primarily on small xenobiotic molecules, other stressors should also be considered such as nanomaterials and nanoplastics which are part of the ‘new entities’ [78]. The focus is currently on the transport of these materials in the body and on their possible toxicity. The induced adaptive pathways concerning these stressors and their costs are less studied at this point.

The adaptive mechanisms described here do not cover all the adaptive mechanisms to the chemical exposome. Epigenetic mechanisms are also implicated, in particular when considering long-term adaptation including multigenerational effects (figure 3). These mechanisms and their contribution to adaptation and/or to possible toxic effects need to be further explored in the future. Furthermore, the role of the microbiome in xenobiotic metabolism, elimination or toxicity is still a matter of active research. The extent to which the microbiome is involved in the adaptation to chemical exposure (i.e. does the microbiome composition change to adapt to the chemical exposure?) and whether this may lead to some side effects is still to be determined.

The extreme diversity of the chemical exposome constitutes a challenge to adaptive pathways. Short-term pathways such as the XMPs (and possibly the microbiome) and longer term pathways involving epigenetic mechanisms are implicated in leading to biological benefits but also to a toxic cost.

## Data Availability

This article has no additional data.
